# HIV-1 drug resistance and associated risk factors in antiretroviral therapy failure: a 2022–2024 surveillance in Huzhou, China

**DOI:** 10.3389/fmicb.2026.1933938

**Published:** 2026-10-29

**Authors:** Zhonghao Lu, Yijie Wu, Yunfeng Zha, Fenfen Dong, Zhongrong Yang, Lei Ji, Xiaojuan Zhu

**Affiliations:** 1 Department of Clinical Microbiology, Huzhou Center for Disease Control and Prevention (Huzhou Municipal Health Inspection and Supervision Agency), Huzhou, Zhejiang, China; 2 Department of Clinical Laboratory, The First People’s Hospital of Huzhou, Huzhou, Zhejiang, China; 3 Department of HIV/TB Control and Prevention, Huzhou Center for Disease Control and Prevention (Huzhou Municipal Health Inspection and Supervision Agency), Huzhou, Zhejiang, China

**Keywords:** acquired drug resistance (ADR), antiretroviral therapy (ART) failure, drug resistance mutations (DRMs), genotypic resistance, HIV-1, molecular epidemiology, risk factors

## Abstract

**Introduction:**

Characterizing acquired drug resistance (ADR) profiles among individuals experiencing antiretroviral therapy (ART) failure is critical for optimizing clinical management.

**Methods:**

This study aimed to delineate the HIV-1 drug resistance landscape, specific resistance-associated mutations, and epidemiological determinants in Huzhou, China, from 2022 to 2024. Blood samples and epidemiological data were collected from 184 HIV-1-infected patients experiencing ART failure. Viral *pol* region sequences were amplified, and drug resistance mutations (DRMs) were interpreted using the Stanford University HIV Drug Resistance Database.

**Results:**

The overall prevalence of ADR was 44.0% (81/184). Resistance to protease inhibitors (PIs) remained notably low at 1.6%,whereas the prevalence of DRMs associated with nucleoside (NRTIs) and non nucleoside reverse transcriptase inhibitors (NNRTIs) reached 27.7% and 41.8%, respectively. The most frequently detected NRTI-associated DRMs included M184V/I and K65R, while dominant NNRTI-associated mutations comprised V179D/E/F, K103N, V106I/L/M, G190A/Q/S/T, and Y181C/L. Furthermore, multivariable logistic regression identified patient age, plasma viral load, and specific HIV-1 subtypes as independent risk factors for DRM development.

**Discussion:**

Ultimately, this study elucidates the evolving mutational landscape among ART-experienced individuals, providing critical evidence-based insights for optimizing personalized antiretroviral regimens and mitigating the onward transmission of drug-resistant HIV-1 variants within the region.

## 1 Introduction

Human immunodeficiency virus (HIV), the etiological agent of acquired immunodeficiency syndrome (AIDS), systematically targets and depletes the host immune system. Untreated infection typically progresses to the clinically advanced stage of AIDS, which is characterized by a high susceptibility to life-threatening opportunistic infections and malignancies (Whiteside, 2011). HIV continues to represent a formidable global public health crisis with pervasive worldwide transmission. To date, the pandemic has accounted for an estimated 91.4 million cumulative infections and 44.1 million deaths attributed to AIDS-related illnesses globally (UNAIDS, 2025). In China, the epidemiological burden remains substantial, with over 1.355 million people living with HIV (PLWH) and 491,000 cumulative reported deaths (National Center for AIDS/STD Control and Prevention, China CDC, 2025). The national incidence of AIDS has exhibited a dynamic trajectory, surging from 0.37 cases per 100,000 population in 2002 to a peak of 10.27 in 2018, before declining to 8.41 per 100,000 in 2021 (Dou et al., 2023). Although current public health interventions have achieved measurable success in curbing new infections, the HIV epidemic persists as a severe and ongoing public health challenge.

As the current cornerstone of HIV management, highly active antiretroviral therapy (HAART) represents the most efficacious strategy for potently suppressing viral replication, significantly reducing disease-specific mortality, and substantially enhancing the health-related quality of life for infected individuals. Recognizing the profound clinical and public health benefits of antiretroviral therapy (ART), China introduced the landmark “Four Frees and One Care” policy in 2002 to ensure the provision of free antiretroviral treatment. Building upon this foundational initiative, the nation comprehensively implemented the 13th 5-Year Action Plan and the “Six Major Projects” aimed at curbing HIV transmission. Consequently, both ART coverage and treatment success rates (virological suppression) have surpassed the 90% threshold. Furthermore, both the absolute number of reported HIV-related deaths and the overall mortality rate have exhibited a continuous, year-over-year decline since 2020. Collectively, these strategic national public health interventions have achieved monumental success in ensuring equitable access to essential care and sustained therapeutics for people living with HIV (PLWH) across China (Yang et al., 2014).

According to the World Health Organization (WHO), epidemiological data from reporting countries between 2014 and 2020 revealed that the prevalence of pretreatment drug resistance (PDR) to efavirenz (EFV) or nevirapine (NVP) exceeded 10% among the vast majority of adults initiating antiretroviral therapy. Strikingly, among adult cohorts experiencing virological failure on NNRTI-based first-line regimens, resistance to EFV or NVP surged to over 90% (World Health Organization, 2021). The scaled-up rollout of antiretroviral medications, synergized with the highly error-prone nature of HIV-1 reverse transcription, invariably drives the accumulation of genomic mutations. These genetic alterations abrogate the inhibitory capacity of therapeutic agents, precipitating clinical treatment failure and, consequently, exacerbating both the incidence of new infections and HIV-related mortality. Domestically, regional epidemiological studies across various provinces in China indicate that the prevalence of acquired drug resistance (ADR) among patients failing ART frequently exceeds 50% (Huang et al., 2024; Wei et al., 2022; Li et al., 2024; Duan et al., 2026). Similarly, a comprehensive European cohort study comprising 26,973 people living with HIV (PLWH) from 1981 to 2019 reported an ADR prevalence of 68.5% (Miranda et al., 2022). The unchecked dissemination of these drug-resistant variants into the general population—exacerbated by a lack of timely molecular surveillance among individuals failing therapy—poses a formidable challenge to public health infrastructures. While routine viral load (VL) monitoring combined with genotypic resistance testing for suspected ART failures facilitates the early detection of drug resistance mutations (DRMs), the ongoing evolution of ARV resistance continues to challenge the long-term sustainability and efficacy of global ART programs.

Situated in northern Zhejiang Province, Huzhou serves as a pivotal geographical and economic nexus within the Yangtze River Delta, a region characterized by intense socioeconomic activity and profound demographic mobility. According to data from the Seventh National Population Census, Huzhou encompasses a resident population of 3.367 million. Notably, migrant populations constitute a substantial 25.8% of the total demographic, comprising 762,000 inter-provincial and 109,000 intra-provincial migrants. Against this dynamic demographic backdrop, a comprehensive 15-year cross-sectional surveillance study (2009–2023) conducted in Huzhou revealed a concerning overarching upward trajectory in the reported incidence of HIV/AIDS, averaging 4.53 cases per 100,000 individuals. The epidemiological profile demonstrates a pronounced male predominance, with sexual transmission—particularly among men who have sex with men (MSM)—identified as the primary driver of viral acquisition (Wang et al., 2025).

Therefore, the present study aimed to characterize the landscape of HIV-1 acquired drug resistance and specific resistance-associated mutations among individuals experiencing ART failure in Huzhou, Zhejiang Province, between 2022 and 2024. Furthermore, we sought to elucidate the key demographic and epidemiological determinants driving the emergence of such viral resistance.

## 2 Materials and methods

### 2.1 Study population and sample collection

This is a retrospective, laboratory-based, cross-sectional surveillance study of PLWH experiencing virological failure in Huzhou between 2022 and 2024. Clinical and epidemiological data were extracted corresponding to the exact date of the virological failure blood sample collection. Participants were enrolled based on the following strict inclusion criteria: (1) Diagnosed with HIV-1 infection in Huzhou and currently receiving antiretroviral therapy for more than 6 months; (2) Patients with virological failure caused by viral rebound after previous suppressive therapy are defined as those with a plasma viral load (VL) ≥ 1,000 copies/mL; and (3) availability of eligible whole blood samples collected in ethylene diamine tetraacetic acid (EDTA) tubes.

Blood samples were routinely collected concurrently with the confirmation of virological failure, prior to any subsequent treatment interruption or regimen modification. For patients with multiple instances of virological failure during the study period, only the sequence from the initial successful amplification was included in the final analysis to avoid duplication. For sample processing, the collected whole blood was centrifuged at 3000 × *g* for 10 min to separate the plasma. The resulting plasma aliquots were immediately cryopreserved at −80° pending downstream molecular analyses.

The study protocol received formal ethical approval from the Medical Ethics Review Committee of the Huzhou Center for Disease Control and Prevention (Approval No.: HZ2024038). All experimental and data collection procedures were conducted in strict adherence to approved institutional guidelines and relevant ethical regulations. Comprehensive sociodemographic characteristics of the participants—encompassing age, sex, transmission route, occupation, and marital status—were systematically extracted from the Chinese Center for Disease Control and Prevention (China CDC) Information System.

### 2.2 HIV-1 RNA extraction and viral gene amplification

Total viral RNA was extracted from the cryopreserved plasma samples using the QIAamp Viral RNA Mini Kit (QIAGEN, Hilden, Germany) in strict accordance with the manufacturer’s protocol. A 1,316-base pair (bp) fragment of the HIV-1 *pol* gene (corresponding to nucleotide positions 2147–3462 of the HXB2 reference genome)—encompassing the entire protease (PR) region and the first 299 amino acid residues of the reverse transcriptase (RT) gene—was amplified utilizing a nested reverse transcription-polymerase chain reaction (RT-PCR) approach.

The first-round RT-PCR was performed using the PrimeScriptTM One Step RT-PCR Kit Ver. 2 (Takara Bio, China). Thermal cycling parameters were configured as follows: reverse transcription at 50° for 30 min and initial denaturation at 94° for 2 min; followed by 30 cycles of denaturation at 94° for 30 s, annealing at 55° for 30 s, and extension at 72° for 150 s; concluding with a final extension at 72° for 10 min. The resulting amplicons subsequently served as templates for the second-round nested PCR using the Ex Taq Kit (Takara Bio, China). The cycling conditions for the nested PCR consisted of an initial denaturation at 94° for 5 min, followed by 30 cycles of 94° for 30 s, 63° for 30 s, and 72° for 150 s, with a final extension step at 72° for 10 min (Zhang et al., 2015).

Successful amplification of the target sequences was verified via 1% agarose gel electrophoresis. The positive PCR products were subsequently sent to Shanghai BioGerm Medical Technology Co., Ltd. (Shanghai, China) for rigorous purification. Bidirectional Sanger sequencing was ultimately performed using overlapping primers on an ABI 3730XL DNA Analyzer (Applied Biosystems, Foster City, CA, USA).

### 2.3 HIV-1 subtyping and genotypic drug resistance profiling

Raw trace files generated from Sanger sequencing were meticulously trimmed and assembled into consensus sequences utilizing Sequencher version 5.4.6 (Gene Codes Corporation, Ann Arbor, MI, USA). The resulting consensus sequences were subsequently aligned and curated using BioEdit version 7.2.0. Initial HIV-1 subtype assignments were performed utilizing the COMET HIV-1 automated online subtyping tool.^1^ To ensure rigorous classification, these preliminary subtype designations were independently cross-verified through phylogenetic analysis; neighbor-joining (NJ) trees were constructed and analyzed using MEGA version 6.0.

Genotypic drug resistance mutations (DRMs) and their corresponding susceptibility profiles were interpreted by leveraging the Stanford University HIV Drug Resistance Database (HIVdb) algorithm version 9.4.1.^2^ The resistance evaluation encompassed 21 commonly prescribed antiretroviral (ARV) agents across three major pharmacological classes. These included seven nucleoside/nucleotide reverse transcriptase inhibitors (NRTIs): abacavir (ABC), zidovudine (AZT), emtricitabine (FTC), lamivudine (3TC), tenofovir disoproxil fumarate (TDF), stavudine (D4T), and didanosine (DDI); five non-nucleoside reverse transcriptase inhibitors (NNRTIs): doravirine (DOR), efavirenz (EFV), etravirine (ETR), nevirapine (NVP), and rilpivirine (RPV); and nine protease inhibitors (PIs): atazanavir/ritonavir (ATV/r), darunavir/ritonavir (DRV/r), lopinavir/ritonavir (LPV/r), fosamprenavir/ritonavir (FPV/r), indinavir/ritonavir (IDV/r), nelfinavir (NFV), saquinavir (SQV), and tipranavir (TPV).

Drug resistance levels were interpreted using the Stanford University HIV Drug Resistance Database (HIVdb) algorithm. Based on the standardized penalty scores, resistance categories were defined as follows: low-level resistance (scores 15–29), intermediate-level resistance (scores 30–59), and high-level resistance (scores ≥ 60). A strict cutoff score of ≥15 was utilized to define the presence of a drug resistance mutation, thereby excluding cases of potential low-level resistance (scores 10–14).

### 2.4 Statistical analysis

All statistical analyses were performed using IBM SPSS Statistics version 27.0 (IBM Corp., Armonk, NY, USA). Continuous variables were expressed as medians with corresponding interquartile ranges (IQRs), whereas categorical data were presented as absolute frequencies and relative percentages. Intergroup comparisons for categorical variables were evaluated utilizing the Pearson χ^2^ test. To systematically elucidate the determinants of HIV-1 drug resistance, we employed a two-step logistic regression framework. Univariable logistic regression analysis was initially conducted to calculate crude odds ratios (ORs) and their 95% confidence intervals (CIs) to assess the strength of preliminary associations. Subsequently, to rigorously account for the effects of potential confounding variables, a multivariable logistic regression model was constructed to estimate adjusted odds ratios (aORs) and 95% CIs. Statistical significance was established at a two-tailed α level of *P* < 0.05.

## 3 Results

### 3.1 Baseline demographic and clinical characteristics of the study population

The overall PCR amplification success rate was 89.5% (197/220). There were no significant systematic differences in baseline demographic characteristics (e.g., age, sex) between the included participants and those excluded due to amplification failure.

An initial cohort of 197 people living with HIV (PLWH) was recruited from Huzhou, northern Zhejiang Province. Following the exclusion of 7 cases due to HIV-1 gene amplification failure and 6 cases lacking comprehensive epidemiological data, a final cohort of 184 eligible participants was retained for downstream analysis. The study population exhibited a pronounced male predominance (81.0%, 149/184), with females accounting for 19.0% (35/184). The median age was 41 years (interquartile range [IQR], 27.5–50; range, 16–73).

Regarding socioeconomic and occupational profiles, the majority of participants were farmers (45.1%, 83/184), followed by enterprise and migrant workers (27.7%, 51/184). The remainder included commercial service personnel (8.2%, 15/184), domestic workers and unemployed individuals (6.5%, 12/184), and other or unspecified roles (12.5%, 23/184). Educationally, a substantial proportion (68.5%) had attained a middle school education or lower. Marital status was primarily categorized as married (42.4%), followed by unmarried (32.6%) and divorced or widowed (25.0%).

Epidemiologically, sexual contact constituted the predominant route of viral acquisition, comprising 67.4% (124/184) heterosexual transmission and 30.4% (56/184) same-sex transmission, whereas injection drug use (IDU) accounted for a marginal 2.2% (4/184). Clinically, the median duration of ongoing ART was 4 years (IQR, 2–7). Baseline immunological and virological assessments revealed a median CD4+ T-cell count of 225.94 cells/μL (IQR, 117.54–367.39) and a median viral load of 18,200 copies/mL (IQR, 3,997.5–52,800). Notably, 44.6% (82/184) of the cohort presented with advanced HIV disease, characterized by severe immunosuppression (CD4+ count ≤ 200 cells/μL). Detailed baseline characteristics of the study population are summarized in Table 1.

**TABLE 1:** Epidemiological characteristics and risk factors associated with the presence of HIV-1 drug resistance mutations (*n* = 184).

Characteristics	Subjects (*N* = 184)	DRM	χ ^2^	*P*	Univariate		Multivariate	
					OR (95% CI)	*P*	OR (95% CI)	*P*
Gender								
Male	149	68	0.83	0.362	1.000			
Female	35	13			0.487 (0.153–1.547)	0.223	–	–
Education								
Illiterate	12	6	0.839	0.933	1.000		1.000	
Primary school	53	25			0.349 (0.069–1.76)	0.202	0.775 (0.199–3.011)	0.713
Junior high school	61	27			0.331 (0.06–1.833)	0.206	0.896 (0.231–3.479)	0.874
Senior high school	38	15			0.123 (0.017–0.9)	0.039	0.632 (0.141–2.827)	0.548
College and above	20	8			0.175 (0.019–1.628)	0.126	0.896 (0.17–4.71)	0.897
Marital status								
Married	78	41	4.074	0.13	1.000			
Divorced or widowed	46	18			0.476 (0.189–1.198)	0.115	–	–
Unmarried	60	22			0.904 (0.305–2.684)	0.856	–	–
Transmission route								
Homosexual transmission	56	26	3.283	0.194	1.000			
Heterosexual transmission	124	55			0.39 (0.151–1.006)	0.052	–	–
Others	4	0			–	–	–	–
Occupation								
Farmer	83	39	1.575	0.813	1.000			
Commercial service worker	15	5			0.469 (0.109–2.012)	0.308	–	–
Domestic service worker and unemployed	12	4			0.683 (0.132–3.527)	0.649	–	–
Factory worker and migrant worker	51	23			1.107 (0.447–2.745)	0.826	–	–
Others and unknown	23	10			1.975 (0.515–7.57)	0.321	–	–
ART duration (years)								
<1	23	9	4.234	0.237	1.000			
1–3	65	29			1.29 (0.391–4.259)	0.676	–	–
4–6	42	14			0.824 (0.221–3.07)	0.773	–	–
>6	54	29			2.497 (0.687–9.073)	0.165	–	–
Age (years old)								
14–24	26	5	7.683	0.021	1.000		1.000	
25–49	108	53			7.719 (1.775–33.561)	0.006	4.681 (1.416–15.477)	0.011
≥50	50	23			6.486 (1.11–37.899)	0.038	4.372 (1.063–17.981)	0.041
Viral load (copies/mL)								
1000–10000	73	42	9.154	0.01	1.000		1.000	
10001–100000	88	30			0.291 (0.124–0.679)	0.004	0.329 (0.163–0.663)	0.002
>100000	23	9			0.609 (0.15–2.479)	0.488	0.432 (0.153–1.225)	0.115
CD4 count (cells/mm3)								
<200	82	37	0.074	0.964	1.000			
200–500	79	34			1.05 (0.446–2.475)	0.911	–	–
>500	23	10			1.611 (0.4–6.48)	0.502	–	–
HIV-1 subtype								
CRF07_BC	70	26	7.961	0.158	1.000		1.000	
CRF01_AE	51	25			3.567 (1.293–9.838)	0.014	2.557 (1.102–5.932)	0.029
CRF08_BC	32	18			3.405 (1.063–10.904)	0.039	2.484 (0.97–6.358)	0.058
B	11	5			1.31 (0.268–6.392)	0.738	1.34 (0.333–5.388)	0.68
URFs	6	4			5.593 (0.652–48.015)	0.117	2.506 (0.384–16.361)	0.337
Others	14	3			0.441 (0.093–2.094)	0.303	0.434 (0.102–1.845)	0.258

### 3.2 Distribution of HIV-1 subtypes and recombinant forms

Phylogenetic analysis of the 184 successfully amplified PR-RT sequences elucidated a highly diverse genetic landscape of HIV-1 within the Huzhou cohort. The circulating viral population was overwhelmingly dominated by circulating recombinant forms (CRFs). Specifically, CRF07_BC emerged as the most prevalent lineage, accounting for 38.0% (70/184) of the isolates. This was followed closely by CRF01_AE (27.7%, 51/184) and CRF08_BC (17.4%, 32/184). In contrast, the pure subtype B represented a comparatively minor fraction of the infections (6.0%, 11/184).

Furthermore, a spectrum of low-frequency CRFs was identified, encompassing CRF67_01B (1.6%), CRF117_0107 (1.1%), CRF68_01B (1.1%), and CRF85_BC (1.1%). Other sporadic CRFs—namely CRF02_AG, CRF22_01A1, CRF55_01B, and CRF80_0107—were rarely detected, collectively comprising only 2.2% (4/184) of the viral pool. Notably, our analysis also revealed the emergence of three distinct unique recombinant forms (URFs), indicating ongoing complex viral recombination in the region. These consisted of URF(B/C) (2.2%), URF(01_AE/C) (1.1%), and URF(07_BC/01_AE) (0.5%).

### 3.3 Prevalence and phenotypic (profiles of HIV-1 acquired drug resistance)

Based on a Stanford HIVdb penalty score cutoff of ≥15, the prevalence of acquired drug resistance (ADR) among the evaluated HIV-1-infected cohort was 44.0% (81/184; 95% confidence interval [CI]: 36.8–51.3%) Stratified by antiretroviral drug classes, the prevalence of drug resistance mutations (DRMs) conferring resistance to protease inhibitors (PIs) was 1.6% (3/184; 95% CI: 0.2–3.5%). In contrast, resistance to nucleoside reverse transcriptase inhibitors (NRTIs) and non-nucleoside reverse transcriptase inhibitors (NNRTIs) was substantially higher, at 27.7% (51/184; 95% CI: 21.2–34.2%) and 41.8% (77/184; 95% CI: 34.7–49.0%), respectively. Regarding multiclass resistance, 23.9% (44/184) of the participants harbored dual-class resistance to NRTIs and NNRTIs, while 1.6% (3/184) exhibited triple-class resistance spanning PIs, NRTIs, and NNRTIs. Bivariate analyses evaluating the associations between baseline demographic/clinical characteristics and the presence of DRMs (Table 1) revealed statistically significant differences only for patient age (χ^2^ = 7.683$, *P* = 0.021) and viral load (χ^2^ = 9.154, *P* = 0.010). No other baseline variables demonstrated statistical significance. The inferred phenotypic resistance levels for specific ARV agents are delineated in Figure 1 (see Supplementary material for detailed information). Among the three individuals harboring PI-associated DRMs, all exhibited varying degrees of resistance to NFV. Notably, one patient demonstrated pan-PI resistance, characterized by high-level resistance specifically to IDV and NFV. Analysis of the NRTI-resistant cases revealed comparable overall resistance rates for ABC, FTC, and 3TC (26.6%, 49/184). However, concerning the magnitude of resistance, a striking 87.8% of the NRTI-resistant cases harbored the M184V/I mutation, responsible for high-level resistance to 3TC and FTC. Resistance to DDI, D4T, and TDF was observed at rates of 18.5%, 14.7%, and 13.6%, respectively, predominantly presenting as intermediate-to-high-level resistance. Within the NNRTI class, remarkably high overall resistance frequencies were noted for EFV (40.2%) and NVP (41.3%), with high-level resistance constituting over 90% of these specific resistant cases. This was followed by DPV (29.9%) and RPV (27.7%), where the proportions of low- and intermediate-level resistance were comparable, though high-level resistance was slightly more prevalent. Finally, resistance to DOR and ETR was primarily intermediate-level, with overall prevalence rates of 23.9% and 19.0%, respectively.

**FIGURE 1:**
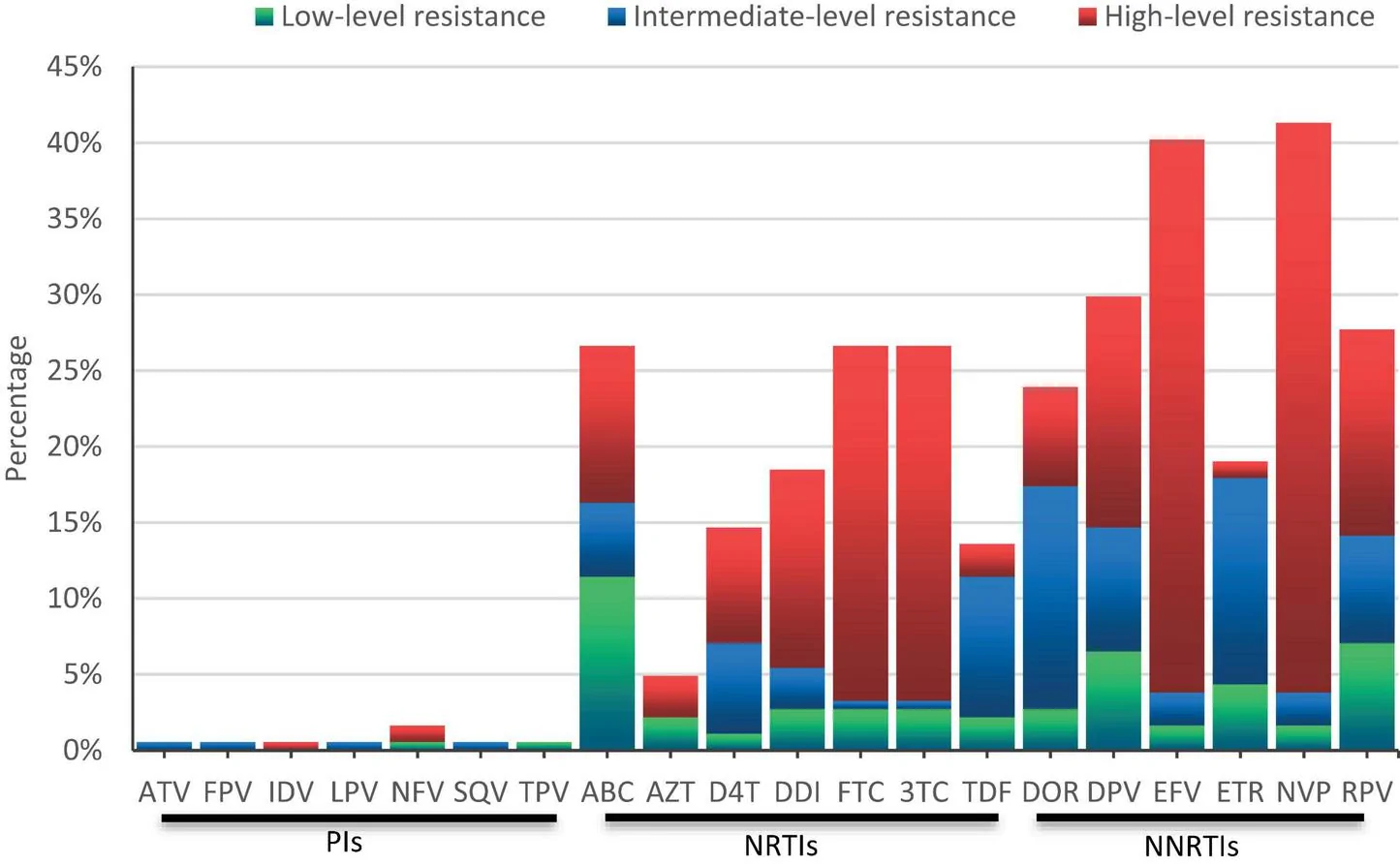
Acquired drug resistance profiles for specific antiretroviral agents in patients failing ART. Resistance categories are defined according to Stanford HIVdb penalty scores: low-level resistance (15–29), intermediate-level resistance (30–59), and high-level resistance (≥60).

### 3.4 Mutational landscape of HIV-1 drug resistance

The specific distributional profile of drug resistance mutations (DRMs) among the evaluated cohort is delineated in Figure 2. Among the 81 viral isolates harboring DRMs, the majority (67.9%, 55/81) exhibited complex mutational patterns conferring resistance to two or more antiretroviral drug classes.

**FIGURE 2:**
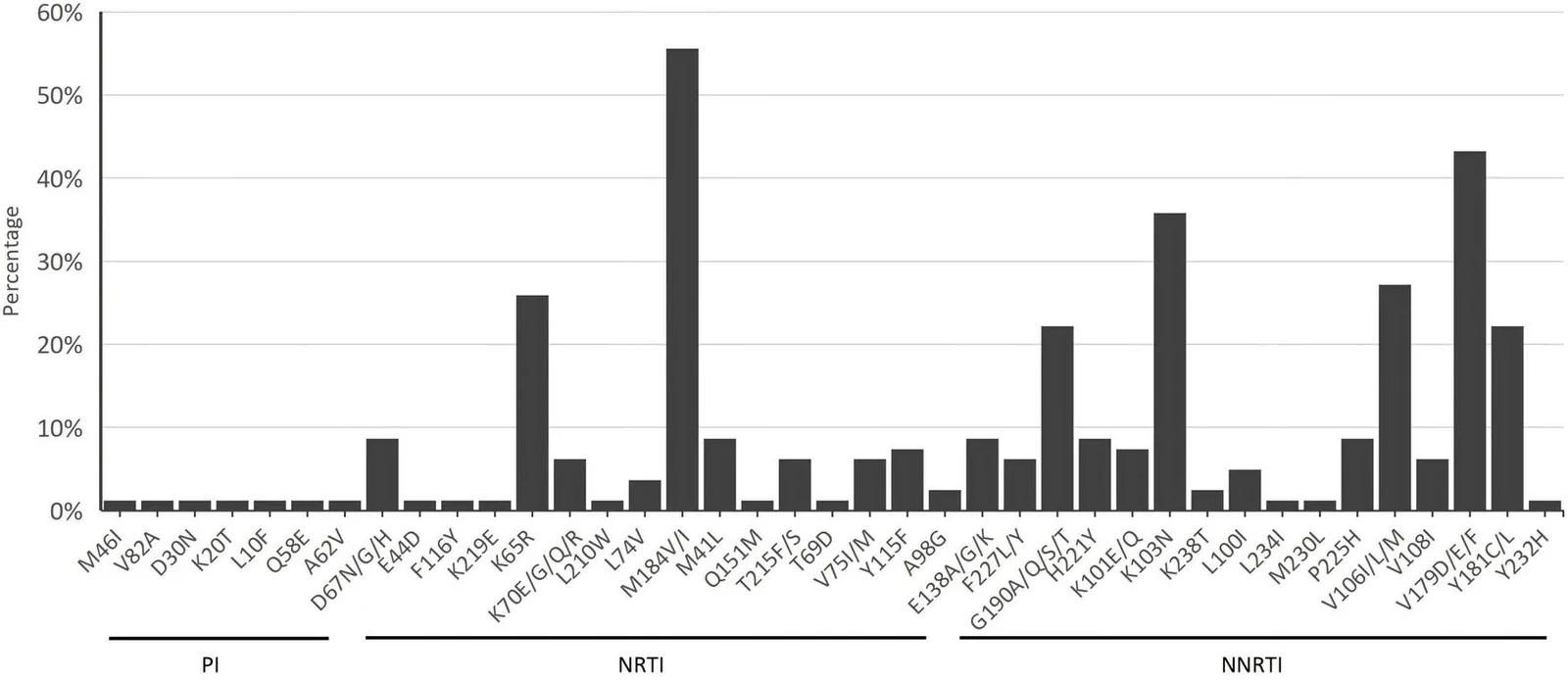
Frequencies of specific HIV-1 genotypic drug resistance mutations (DRMs) among the evaluated cohort.

Regarding PI-associated major DRMs, the combination of M46I + V82A (1.2%, 1/81) and the single D30N mutation (1.2%, 1/81) were identified. Moreover, the co-occurrence of M46I and V82A leads to a substantial decline in susceptibility to protease inhibitors, with Stanford HIVdb pattern scores assigned as 35 for ATV/r and 50 for LPV/r (Stanford University, 2026). Conversely, the D30N substitution specifically confers high-level phenotypic resistance to nelfinavir (NFV).

Within the NRTI-associated mutational spectrum, the most frequently detected variants were M184V/I (55.6%, 45/81), followed by K65R (25.9%, 21/81). For the NNRTI class, the dominant mutational landscape comprised V179D/E/F (43.2%, 35/81), K103N (35.8%, 29/81), and V106I/L/M (27.2%, 22/81), along with the G190A/Q/S/T and Y181C/L variants (both accounting for 22.2%, 18/81).

### 3.5 Epidemiological and clinical factors associated with the detection of HIV-1 drug resistance

Initial univariable logistic regression analysis identified educational attainment, patient age, plasma viral load (VL), and HIV-1 subtype as significant variables associated with the presence of DRMs. Upon incorporating these covariates into a multivariable logistic regression model, patient age, an intermediate VL (10,000–100,000 copies/mL), and HIV-1 subtype retained statistically significant associations with the detection of acquired drug resistance (*P* < 0.05).

Age emerged as the most substantial independent determinant for DRM development. Compared to the youngest demographic cohort (14–24 years), individuals in the intermediate (25–49 years) and older (≥50 years) age brackets exhibited a more than four-fold increased risk of harboring drug-resistant variants.

Regarding virological parameters, an intermediate VL of 10,000–100,000 copies/mL demonstrated a significant inverse association with the presence of DRMs when compared to the lower-tier reference cohort (1,000–10,000 copies/mL). Specifically, patients in this intermediate stratum were significantly less likely to harbor DRMs, presenting only 32.9% of odds observed in the reference group. Furthermore, viral genetics exerted a profound impact: infections driven by the CRF01_AE lineage carried a more than two-fold higher likelihood of harboring DRMs compared to those caused by the predominant CRF07_BC subtype.

## 4 Discussion

In China, VF is often defined as defined as the persistence of plasma HIV-1 RNA levels ≥ 1,000 copies/mL following at least 6 months of continuous antiretroviral therapy (ART). Longitudinally, unresolved VF frequently serves as the direct prognostic precursor to the relentless accumulation of drug resistance mutations (DRMs). In the present cohort from Huzhou, the overall prevalence of acquired drug resistance (ADR) among patients experiencing VF was 44.0%. A granular dissection of this resistance landscape revealed that resistance to NRTIs and NNRTIs stood at 27.7% and 41.8%, respectively, with a striking 23.9% of the cohort harboring dual-class resistance.

This regional ADR prevalence (44.0%) is comparable to the rate reported in Zhongwei, Ningxia (40.0%; Duan et al., 2026) and aligns with profiles observed in other Chinese regions (e.g., Guangzhou, 46.1%; Lv et al., 2025), while remaining lower than the rates reported in certain international settings, such as Ethiopia (77.8%; Getaneh et al., 2022). By stark contrast, in well-resourced regions like Europe, the synergistic implementation of proactive genotypic surveillance and the widespread adoption of single-tablet regimens (STRs) characterized by high genetic barriers to resistance have successfully curtailed both the incidence of VF and the subsequent emergence of ADR (Schultze et al., 2015).

At the molecular level, genotypic resistance profiling revealed that the most prevalent NRTI-associated mutations within our cohort were M184V/I (55.6%), K65R (25.9%). Although the M184V/I substitution confers high-level resistance to lamivudine (3TC) and emtricitabine (FTC), it induces hypersusceptibility to tenofovir (TDF) and zidovudine (AZT) (Mimtsoudis et al., 2024). Consequently, in the design of clinical salvage regimens, 3TC or FTC is frequently retained despite the presence of M184V/I; this strategy exerts continuous virological selective pressure to maintain the virus in a state of diminished replicative fitness. K65R, recognized as the signature TDF resistance mutation, mediates intermediate-to-high-level cross-resistance encompassing TDF, abacavir (ABC), and didanosine (DDI). Many S68G (19.8%) substitutions were also identified in this study. Conversely, the S68G substitution does not independently augment drug resistance; it functions fundamentally as a compensatory mutation to restore the viral replicative capacity compromised by the K65R variant (Li et al., 2020).

Notably, we identified a rare case harboring the Q151M complex (Q151M + A62V + V75I + F116Y). This unique reverse transcriptase mutational pattern, when synergized with these secondary accessory substitutions, confers high-level multi-nucleoside resistance (MNR) against virtually all canonical NRTIs (including AZT, ABC, TDF, 3TC, and FTC). Such a profile effectively obliterates the efficacy of the entire NRTI backbone, critically necessitating the deployment of integrase strand transfer inhibitors (INSTIs) or high-barrier PIs to construct viable salvage therapeutic regimens (Das et al., 2017).

Within the NNRTI mutational spectrum, V179D/E exhibited the highest detection frequency. Operating as a polymorphic accessory mutation, it marginally attenuates viral susceptibility across the NNRTI class. However, the synergistic combination of V179F and Y181C compromises susceptibility to etravirine (ETR) and rilpivirine (RPV) (Stanford University, 2026). Furthermore, the high prevalence of K103N, V106I/L/M, and G190A reduces the efficacy of efavirenz (EFV) and nevirapine (NVP). In addition, E138 substitutions (particularly E138A/K/G) have demonstrated an increased prevalence in recent surveillance. The E138 locus is a critical resistance determinant not only for first-generation NNRTIs but also for second-generation agents (e.g., RPV and ETR). Emerging evidence indicates that the CRF55_01B subtype harbors a high background frequency of both natural and acquired E138G polymorphisms, posing a latent clinical threat to the future widespread rollout of RPV-based regimens in endemic regions (Zhou et al., 2022).

Acquired resistance to PIs remains clinically rare, corroborated by multiple national cohort studies reporting prevalence rates below 3%. Nevertheless, the atypical Q58E mutation warrants distinct clinical attention. Within the CRF07_BC lineage, Q58E frequently manifests as a naturally occurring polymorphism, with a background prevalence reaching 8–10% (Zhang et al., 2024). While an isolated Q58E substitution is insufficient to confer high-level resistance against potent PIs like lopinavir/ritonavir (LPV/r), its presence underscores the critical imperative to factor baseline viral polymorphisms into regimen selection across different endemic subtypes. This vigilance is essential to preempt the induction of complete clinical resistance under prolonged, low-level pharmacological selective pressure. Moreover, the synergistic interaction between M46I and V82A precipitates a broad-spectrum decline in susceptibility to protease inhibitors (Stanford University, 2026).

Multivariable analysis confirmed an association between patient age, plasma viral load (VL), and HIV-1 subtype with the presence of drug resistance. The higher prevalence of resistance observed among older individuals aligns with broader demographic trends in China, where individuals aged ≥50 years accounted for 48.1% of newly diagnosed infections in 2022. Epidemiologically, this association in older cohorts is primarily attributable to a longer cumulative duration of antiretroviral therapy and potential historical exposure to earlier ART regimens with lower genetic barriers to resistance. Additionally, age-related polypharmacy and potential pharmacokinetic variations may contribute to suboptimal drug exposure in older populations (Cao et al., 2024).

From a viral evolutionary perspective, recent phylogenetic evidence indicates that the CRF01_AE lineage exhibits an intrinsically accelerated evolutionary rate (2.08 × 10^–3^ substitutions/site/year). Notably, even in antiretroviral-naive states, this specific subtype harbors a pronounced background prevalence of natural baseline polymorphisms, including S68G and V179D/E (Minna et al., 2025). In our cohort, we also observed a higher DRM detection rate among patients infected with the CRF01_AE subtype compared to those with the CRF07_BC subtype. However, this finding may also be influenced by unmeasured potential confounding factors, such as disparities in local transmission networks across populations, variations in prior baseline characteristics, or differences in specific treatment adherence patterns.

Several limitations of this study warrant acknowledgment. First, lacking pre-treatment baseline genotypes, we could not entirely exclude the confounding effect of transmitted drug resistance (TDR), which has an estimated prevalence exceeding 10% in China (Hao et al., 2025). Second, detailed adherence metrics and granular antiretroviral treatment histories were not fully available in this retrospective cohort. Because poor adherence can independently drive virologic failure without selecting for resistance, our multivariable analysis identifies statistical associations with the detection of DRMs, rather than independent causal risk factors for DRM development.

Additionally, our methodological scope was limited. Resistance profiling did not encompass integrase strand transfer inhibitors (INSTIs), which may result in an underestimation of the evolving resistance landscape as INSTI-anchored regimens become standard. Finally, our reliance on conventional Sanger sequencing inherently restricts the detection of low-abundance drug-resistant variants (<20% of the viral quasispecies). Future integration of next-generation sequencing (NGS) could improve diagnostic sensitivity for these minor variants (Ouyang et al., 2024) and further refine regional surveillance strategies.

## 5 Conclusion

While the predominance of NRTI and NNRTI resistance mutations reflects known failure patterns of standard first-line regimens, this study provides the first systematic, contemporary baseline data on acquired drug resistance in Huzhou. Ultimately, these virological and epidemiological insights go beyond confirming known resistance patterns; they provide essential local evidence to inform regional drug resistance surveillance and justify programmatic transitions toward more robust, INSTI-based clinical monitoring strategies in this region.

## Data Availability

The raw sequences generated in this study have been deposited in the NCBI GenBank database under accession numbers QB139180–QB139363.

## Footnotes

1
https://comet.lih.lu

2
https://hivdb.stanford.edu/
